# Mechanism of the Rpn13-induced activation of Uch37

**DOI:** 10.1007/s13238-014-0046-z

**Published:** 2014-04-22

**Authors:** Lianying Jiao, Songying Ouyang, Neil Shaw, Gaojie Song, Yingang Feng, Fengfeng Niu, Weicheng Qiu, Hongtao Zhu, Li-Wei Hung, Xiaobing Zuo, V. Eleonora Shtykova, Ping Zhu, Yu-Hui Dong, Ruxiang Xu, Zhi-Jie Liu

**Affiliations:** 1 National Laboratory of Biomacromolecules, Institute of Biophysics, Chinese Academy of Sciences, Beijing, 100101 China; 2 iHuman Institute, ShanghaiTech University, Shanghai, 201210 China; 3 Shandong Provincial Key Laboratory of Energy Genetics, Qingdao Institute of Bioenergy and Bioprocess Technology, Chinese Academy of Sciences, Qingdao, 266101 China; 4 Physics Division, Los Alamos National Laboratory, Los Alamos, NM 87545 USA; 5 X-ray Science Division, Argonne National Laboratory, 9700 South Cass Avenue, Argonne, IL 60439 USA; 6 Institute of Crystallography, Russian Academy of Sciences, 59 Leninsky Pr., 117333 Moscow, Russian Federation; 7 Center for Multi-disciplinary Research, Institute of High Energy Physics, Chinese Academy of Sciences, Beijing, 100049 China; 8 Department of Nerosurgery, The Military General Hospital of Beijing PLA, Beijing, 100700 China

**Keywords:** Uch37-Rpn13 complex, de-ubiquitination, SAXS analysis, oligomerization, iso-peptidase

## Abstract

Uch37 is a de-ubiquitinating enzyme that is activated by Rpn13 and involved in the proteasomal degradation of proteins. The full-length Uch37 was shown to exhibit low iso-peptidase activity and is thought to be auto-inhibited. Structural comparisons revealed that within a homo-dimer of Uch37, each of the catalytic domains was blocking the other’s ubiquitin (Ub)-binding site. This blockage likely prevented Ub from entering the active site of Uch37 and might form the basis of auto-inhibition. To understand the mode of auto-inhibition clearly and shed light on the activation mechanism of Uch37 by Rpn13, we investigated the Uch37-Rpn13 complex using a combination of mutagenesis, biochemical, NMR, and small-angle X-ray scattering (SAXS) techniques. Our results also proved that Uch37 oligomerized in solution and had very low activity against the fluorogenic substrate ubiquitin-7-amino-4-methylcoumarin (Ub-AMC) of de-ubiquitinating enzymes. Uch37Δ^Hb,Hc,KEKE^, a truncation removal of the C-terminal extension region (residues 256–329) converted oligomeric Uch37 into a monomeric form that exhibited iso-peptidase activity comparable to that of a truncation-containing the Uch37 catalytic domain only. We also demonstrated that Rpn13C (Rpn13 residues 270–407) could disrupt the oligomerization of Uch37 by sequestering Uch37 and forming a Uch37-Rpn13 complex. Uch37 was activated in such a complex, exhibiting 12-fold-higher activity than Uch37 alone. Time-resolved SAXS (TR-SAXS) and FRET experiments supported the proposed mode of auto-inhibition and the activation mechanism of Uch37 by Rpn13. Rpn13 activated Uch37 by forming a 1:1 stoichiometric complex in which the active site of Uch37 was accessible to Ub.

**Electronic supplementary material:**

The online version of this article (doi:10.1007/s13238-014-0046-z) contains supplementary material, which is available to authorized users.

## Introduction

**Figure 1:**
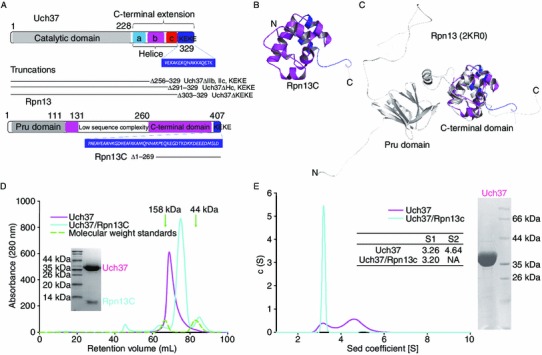
**Oligomerization of Uch37 in solution**. (A) Diagrammatic representation of the constructs of Uch37 and Rpn13 used in this study. (B) Cartoon representation of the structure of Rpn13C solved by NMR. The color representation of Rpn13 is shown as the same as Fig. 1A. (C) Overlay of the cartoon of the NMR structure of Rpn13C (AA 270–407) over the NMR structure of the full-length Rpn13 (grey color; PDB ID 2KR0). The truncation had no effect on the folding of the protein. (D) Size exclusion profiles revealed that Uch37 has a shorter retention time than the Uch37-Rpn13C complex. Proteins from the peaks were analyzed by SDS-PAGE (inset). Ovalbumin (44 kDa) and γ-globulin (158 kDa) were run as standards under identical conditions. The positions of the standards are marked with green arrows. (E) Analytical ultracentrifugation (AUC) analysis of Uch37 and the Uch37-Rpn13C complex. Uch37 was heterogeneous and exhibited two distinct peaks, whereas the Uch37-Rpn13C complex was homogenous. The sedimentation co-efficient (S value) calculated for Uch37 and the Uch37-Rpn13C complex are shown in the inset

The 26S proteosome is a large, modular, multi-protein complex composed of the 20S catalytic core particle (CP) and the 19S regulatory particle (RP). The barrel-shaped 20S CP is associated with the 19S RP in a 1:2 stoichiometry. Prior to degradation, the target protein is first de-ubiquitinated. The RP module is responsible for the recognition of the ubiquitin (Ub) chain and for its subsequent de-ubiquitination. The 19S RP can recognize Ub using at least two Ub receptors—Rpn10 and Rpn13. Rpn10 (also called PMSD5 and S5A) contains two Ub-interacting motifs (UIMs), which display selectivity for longer poly-Ub chains (Young et al., 1998; Glickman et al., 2009). In contrast, Rpn13 (also called ADRM1) is a recently identified Ub receptor comprising 407 amino acids (AAs), with an N-terminal pleckstrin-like receptor for the Ub (Pru) domain (AA 22–130; also known as the ubiquitin-binding domain, or UBD) that can bind K48-linked di-Ub (Groll et al., 2008; Husnjak et al., 2008) and a C-terminal domain (AA 253–407; i.e., Uch37-binding domain) (Fig. 1A). Once there is recognition, the process of de-ubiquitination is initiated by de-ubiquitinating enzymes (DUBs), such as Rpn11 (also called POH1 and PMSD14), Usp14, and Uch37 (Reyes-Turcu et al., 2009). Together, these DUBs disassemble the poly-Ub chain and recycle the ubiquitin during proteasomal degradation. After de-ubiquitination, the protein is partially unfolded and translocated into the cavity of the 20S CP for degradation via a threonine-dependent nucleophilic attack (Voges et al., 1999; Zhu et al., 2005; Liu et al., 2006).

Ubiquitin C-terminal hydrolases (UCHs) release active Ub from Ub adducts or precursors and have been shown to play important roles in cellular events, such as DNA repair, protein degradation, and modulation of signaling pathways. At least four different types of UCHs are known to exist in human beings-UCH-L1, UCH-L3, UCH-L5, and BAP1. These enzymes have a highly conserved catalytic core (UCH) domain consisting of approximately 220 residues. The crystal structures of the UCH domain for UCH-L1, UCH-L3, and YUH1 (UCH homolog in yeast) are known (Johnston et al., 1997; Johnston et al., 1999; Misaghi et al., 2005; Das et al., 2006). Recently, structures of the UCH catalytic domain and the full-length Uch37 were also reported (Nishio et al., 2009; Burgie et al., 2011; Maiti et al., 2011). The topology and overall configuration of the UCH domain are similar in all hydrolases. In addition, all UCHs appear to contain a flexible active site cross-over polypeptide loop that regulates entry of the substrate into the active site and to impart its specificity (Popp et al., 2009; Zhou et al., 2012).

Human ubiquitin C-terminal hydrolase Uch37 (also called ubiquitin carboxy-terminal hydrolase isozyme L5, UCH-L5) is a 37 kDa de-ubiquitinating enzyme composed of an N-terminal UCH domain and C-terminal extension region (Fig. 1A). Uch37 progressively removes Ub from the distal part of the poly-Ub chain, which may rescue poorly ubiquitinated proteins from proteolysis (Lam et al., 1997; Koulich et al., 2008). The C-terminal extension region of Uch37 plays an important role in several protein-protein interactions. For example, Smad7 acts as an adaptor molecule to recruit Uch37 to the type I TGF-β (transforming growth factor-β) receptor, where Uch37 up-regulates TGF-β-dependent gene expression by de-ubiquitinating and stabilizing the type I TGF-β receptor (Wicks et al., 2005; Ventii and Wilkinson, 2008). The Ino80 complex, an ATP-dependent chromatin remodeling complex, associates with and recruits Uch37 to the nucleosome during transcription and DNA repair by NFRKB (Yao et al., 2008). Another important protein-protein interaction of Uch37 that has been studied extensively is its association with Rpn13. The full-length Uch37 exhibits low iso-peptidase activity against Ub-AMC (ubiquitin-7-amino-4-methylcoumarin) (Yao et al., 2006). Rpn13 can interact with Uch37 and recruit it to the proteasome via its C-terminal 46 residues (also called the KEKE motif) and activates Uch37. The de-ubiquitination activities of this activated Uch37 have been shown to be higher than those of the UCH catalytic domain alone (Hamazaki et al., 2006; Qiu et al., 2006; Yao et al., 2006). Interestingly, a truncation of Uch37 containing the UCH core domain alone exhibited activity comparable to that of its homolog UCH-L3 (Yin et al., 2011), suggesting that the C-terminal extension region likely plays an important role in the auto-inhibition of Uch37. Furthermore, whereas the Rpn13-Uch37 complex can hydrolyze only small adducts of ubiquitin, such as Ub-AMC, a Rpn13-Uch37 complex that is incorporated on 19S RP can process large ubiquitin conjugates, such as di-ubiquitin (Yao et al., 2006).

The C-terminus of Uch37 might contain clues that explain how the full-length Uch37 remains auto-inhibited whereas the isolated UCH domain of the same protein exhibits activity. As discussed by Burgie et al., the Uch37 tetramer observed in the crystal structure could impose auto-inhibition in which the oligomeric assembly of Uch37 sterically precludes ubiquitin binding (Burgie et al., 2011). The C-terminus of Rpn13 has been shown to specifically bind to and activate Uch37 (Hamazaki et al., 2006; Qiu et al., 2006; Yao et al., 2006). However, the mechanism by which Rpn13 activates Uch37 is not known. To gain a clear understanding of the auto-inhibition mechanism of Uch37 and the Uch37 activation by Rpn13, we investigated the Uch37-Rpn13 complex using a combination of mutagenesis, biochemical, NMR, and SAXS analyses. Initially, a role for the C-terminal region of Uch37 in the oligomerization of the protein was established by characterizing the relationship between the oligomerization states of several C-terminal truncations of Uch37 and their activities. These results were compared with the oligomerization state and stoichiometry of the Uch37-Rpn13 complex in solution to determine whether a change in the oligomerization state of the protein results in the activation of Uch37. These data coupled with the SAXS analysis of Uch37 alone and its complex with Rpn13 in solution provided the structural basis for the auto-inhibition of Uch37 in solution. A mechanism for the activation of Uch37 by Rpn13 is discussed.

## Results and Discussion

### NMR structure of the Rpn13 C-terminal domain

Our initial attempts at crystallizing the full-length Rpn13 failed. Analysis of Rpn13 using secondary structure prediction programs suggested the presence of an approximately 130-AA-long loop connecting the Pru domain to the C-terminal domain. This prediction was later confirmed by NMR models of the full-length Rpn13 (Chen et al., 2010). Furthermore, it is known that the Rpn13 C-terminal domain can interact with Uch37 via its KEKE motif and activate Uch37 in a manner similar to that of the full-length Rpn13. Using this information, we designed seven Rpn13 C-terminal constructs with domain boundaries encompassing residues 211–407, 230–407, 253–407, 259–407, 270–407, 283–407, and 297–407. One of these constructs, AA 270–407 (hereafter referred to as Rpn13C) (Fig. 1A), was sufficiently stable when kept at 25°C. Therefore, we used this Rpn13C for further analysis of the Rpn13-Uch37 complex.

To determine whether the truncation had any effect on the overall structure of Rpn13, we solved the structure of Rpn13C using NMR independently (Fig. 1B) and compared it to the structures of the full-length Rpn13 (PDB ID 2KR0) and the AA 253–407 fragment (PDB ID 2KQZ) solved by Chen et al. (Chen et al., 2010). The statistics of the final model are listed in Supplementary Table S1. The NMR structure of Rpn13C was similar to that of the same region in the full-length protein (PDB ID 2KR0; Fig. 1B and 1C), except for some minor deviations of a few N-terminal residues. Because this truncation did not appear to distort the overall structure of the protein, Rpn13C was used for the subsequent studies.

### Oligomerization state analysis of Uch37 alone and its complex with Rpn13

Preliminary size exclusion chromatography (SEC) studies of Uch37 and its complex with Rpn13C revealed that at a concentration of approximately 10 mg/mL, Uch37 had a smaller retention volume than the Uch37-Rpn13C complex (Fig. 1D). This finding was unexpected and provided the first indication that the oligomerization of Uch37 might be different between Uch37 alone and the Uch37-Rpn13C complex. To determine the oligomerization state of Uch37 under solution conditions, we injected different concentrations of Uch37 into a 120-mL Superdex G200 SEC column and estimated the MWs of the proteins from their elution volumes using a standard curve. The MWs observed for Uch37 at 0.4, 1.6, and 10 mg/mL were 50.7, 58, and 128.9 kDa, respectively (Table 1 and Fig. S1). These results indicated a concentration-dependent oligomerization of Uch37, which is consistent with the previous report by Burgie et al. (Burgie et al., 2011). In sharp contrast, the elution volume of the Uch37-Rpn13C complex was independent of the concentration. The complex eluted at 77.92 and 77.84 mL at concentrations of 1.2 and 8 mg/mL, respectively. The MWs for these elution volumes were 66.4 and 66.8 kDa, respectively (Table 1). The exact oligomerization state of Uch37-Rpn13C complex could not be determined from the SEC profile because the theoretical MW of the complex calculated from its primary amino acid sequence is approximately 52.6 kDa. The SDS-PAGE analysis of the protein from the peak indicated the presence of both Uch37 and Rpn13C. Therefore, Uch37 and Rpn13C likely formed a stable complex. We performed analytical ultracentrifugation (AUC) analysis to further confirm the MW and to obtain the oligomerization state of the Uch37-Rpn13C complex. Sedimentation velocity experiments revealed a single, sharp peak with an S value of 3.20 corresponding to a MW of approximately 56.4 kDa (Fig. 1E). This MW, which is close to the theoretical MW (52.6 kDa) of the Uch37-Rpn13C complex, suggested that the Uch37-Rpn13C complex existed as a heterodimer in solution. A frictional ratio of 1.577 (f/f0 > 1.2) indicated that the Uch37-Rpn13C complex adopted an elongated shape in solution (Supplementary Table S2).

**Table 1:** Molecular weights and oligomerization states of Uch37 and its complex with Rpn13C estimated by different methods

Protein	MW (kDa) calculated from primary sequence	MW estimated from different methods			Oligomerization status
		Method	Concentration (mg/mL)	MW (kDa)	
Uch37	37.0	SEC	0.4	50.7	Monomer, dimer
			1.6	58.0	Monomer, dimer
			10.0	128.9	Trimer, tetramer
		AUC	~1.0	28.8 and 50.1	Monomer, dimer
		SAXS	1.3	117.7	Dimer, trimer, tetramer
Uch37-Rpn13C	52.5	SEC	1.2	66.4	Monomer
			8.0	66.8	Monomer
		AUC	~1.0	56.4	Monomer
		SAXS	1.3	64.6	Monomer
Uch37Δ^Hb,Hc,KEKE^	29.1	SEC	>7.0	34.3	Monomer
Uch37Δ^Hc,KEKE^	33.3	SEC	>4.0	38.0	Monomer
		AUC	~1.0	30.1	Monomer
Uch37Δ^KEKE^	34.9	SEC	>1.5	41.9	Monomer

SEC, size exclusion chromatography; AUC, analytical ultracentrifugation; SAXS, small angle X-ray scattering

**Figure 2:**
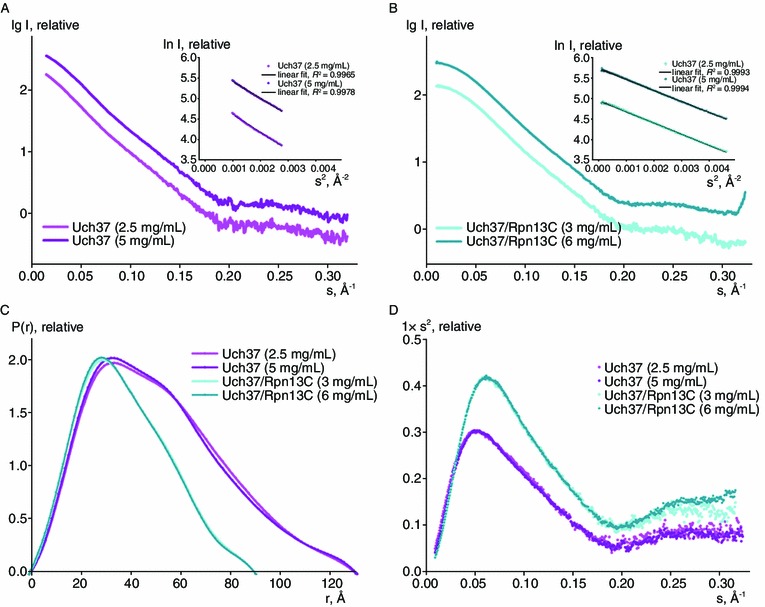
**SAXS analysis of Uch37 and Uch37-Rpn13C**. The scattering curves for Uch37 (A) and the Uch37-Rpn13C complex (B) generated from different concentrations of the protein were similar. The P(r) distribution function (C) and Kratky plots (D) of Uch37 and the Uch37-Rpn13C complex were calculated from the experimental data. The plots suggested that there was neither any concentration-dependent aggregation nor any radiation damage during the SAXS data collection

Figure 2 presents the SAXS data collected for different concentrations of Uch37 and the Uch37-Rpn13C complex, and Table 2 lists the parameters derived from those curves. The Guinier plots clearly indicated that no discernible aggregation or radiation damage occurred during the data collection (Figs. 2A and 1B). The P(r) curve of Uch37 contained a major peak at 38 Å and a small shoulder at 58 Å, with a D_max_ of 125 Å, indicating that Uch37 assumed an elongated conformation in solution. Alternatively, Uch37 could be more compact when associated with Rpn13C (Fig. 2C). The Kratky plots of both Uch37 and the Uch37-Rpn13C complex exhibited a clear peak with plateaus (Fig. 2D). This finding suggested that both Uch37 and the Uch37-Rpn13C complex were well folded and contained some flexible regions. The MWs calculated from the SAXS P(r) functions for Uch37 and Uch37-Rpn13C complex were approximately 117.7 and 64.6 kDa, respectively. When compared to the theoretical MWs (Uch37, 37 kDa; Uch37-Rpn13C complex, 52.6 kDa), the SAXS-based MW estimations further highlighted the ability of Uch37 to form oligomers, although the Uch37-Rpn13C complex might exist as a heterodimer.

**Table 2:** **Parameters derived from SAXS curves**

Protein sample	R_g_ (Å)^a^	D_max_ (Å)^b^	V_Porod_ (Å^3^)^c^	MW^d^ (kDa)	Oligomeric state
Uch37	40.74 ± 0.14	120–130	197,367 ± 12,025	117.7 ± 1.1	Dimer and tetramer
Uch37-Rpn13C	29.73 ± 0.17	85–90	90,879 ± 1403	64.6 ± 1.0	Heterodimer

^a^The real-space R_g_ was estimated by calculating P(r) using GNOM

^b^D_max_ was estimated by calculating P(r) using GNOM

^c^The Porod volume was calculated using PRIMUS

^d^The molecular weights (MWs) were predicted using the web server http://www.if.sc.usp.br/~saxs/saxsmow.html) (Fischer et al., 2010)

^e^Values are the average of three independent experiments; error bars represent the standard error of the mean

Taken together, the SEC, AUC, and SAXS analyses of different concentrations of Uch37 suggested that Uch37 could exist as a mixture of different oligomeric states in solution and Uch37 was de-oligomerized in the presence of Rpn13, forming a stable heterodimer of Rpn13 and Uch37. However, the factors that trigger the oligomerization of Uch37 inside the cell are currently unclear. In addition, Burgie et al. discussed the factors that could cause molecular crowding of Uch37 and its subsequent oligomerization (Burgie et al., 2011).

### Oligomerization of Uch37 and its de-oligomerization by Rpn13

We used a known crystal structure of Uch37 (PDB ID 3IHR) (Burgie et al., 2011) to gain information about the mode of oligomerization of Uch37 using PDBePISA (http://www.ebi.ac.uk/msd-srv/prot_int/) (Krissinel and Henrick, 2007), a program that determines the oligomerization state of a protein from its crystal structure. Inspection of the symmetry mates revealed that two Uch37 molecules formed extensive hydrogen bonds and salt bridges at the N-terminal core domain, and the major contact area spanned 1,831.2 Å^2^ and was 11.1% of the surface area of each monomer. The solvation free energy gained upon formation of the interface (Δ^i^G) was calculated to be 6.8 kcal/mol. Two such dimers then assembled into a tetramer through interactions involving the C-terminal extension region (Fig. S2A and S2B). The solvent-accessible surface area of monomeric units buried upon formation of the tetrameric assembly was 12,600 Å^2^. This analysis suggests that Uch37 most likely forms and exists as a dimer or tetramer in solution. Interestingly, this type of analysis using the structure of the Uch37 catalytic domain (PDB ID 3A7S) indicated that it could not form a dimer or tetramer. However, the possibility that Uch37 forms other oligomeric states when other factors are present in the solution cannot be ruled out.

**Figure 3:**
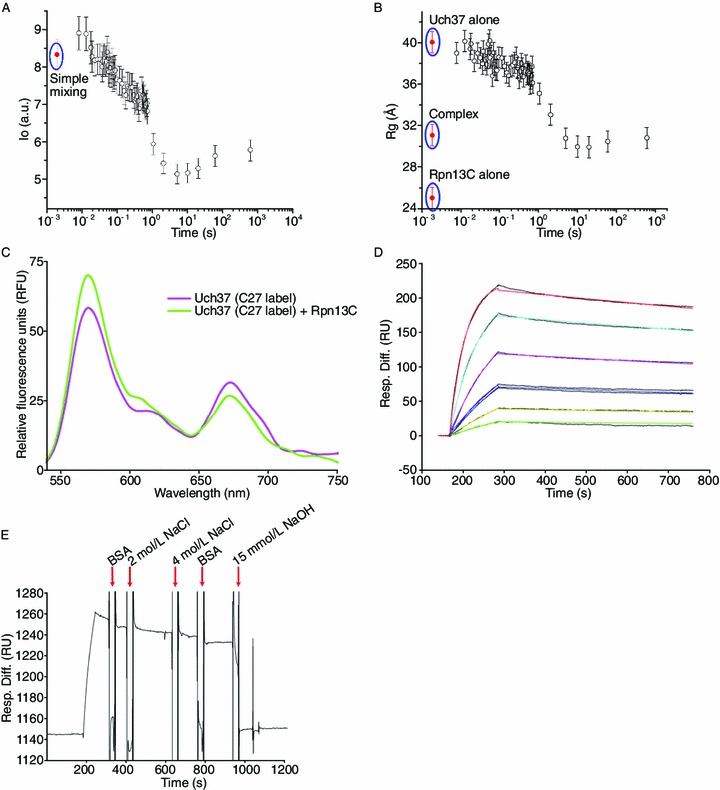
**TR-SAXS, FRET, and SPR analysis of the Uch37-Rpn13C complex**. (A) Graph of scattering intensity (I_0_) versus time and radius of gyration (R_g_) versus time (B) during time-resolved SAXS analysis of a mixture of Uch37 and Rpn13C. Simple mixing represents the scenario where samples are assumed to be physically mixed without any chemical reaction taking place between the samples. The I_0_ and R_g_ values of Uch37 alone, Rpn13C alone, and the Uch37-Rpn13C complex are shown as red dots. The error bars represent the standard error of the mean of three independent measurements. (C) For the FRET experiments, three out of four cysteines present in Uch37 were mutated to alanine. Cys27 of Uch37 was conjugated to Alexa Fluor 555 (absorption maxima at 555 nm; emission maxima at 565 nm) and Alexa Fluor 647 (absorption maxima at 650 nm; emission maxima at 668 nm) (referred to as C27-label) randomly according to the manufacturer’s instructions. For all emission scans, the fluorophores in solution were excited at 550 nm, and their fluorescence emission was measured from 525 to 750 nm. The FRET experiment illustrating the decrease in the acceptor fluorescence at 664 nm and the corresponding increase in the donor fluorescence at 553 nm upon the addition of Rpn13C suggests reversal of the Uch37 oligomerization. (D, E) SPR analysis of the binding of Uch37 to Rpn13C. Rpn13C was immobilized, whereas a gradient of Uch37 was applied to quantify the binding affinity (panel D). Different colors indicate different concentrations of Uch37: green (15.6 nmol/L), yellow (31.2 nmol/L), blue (62.5 nmol/L), pink (125 nmol/L), cyan (250 nmol/L), and red (500 nmol/L). The binding was strong, and neither BSA (10 mg/mL) nor NaCl (4 mol/L) could break the complex. The proteins could be separated using 15 mmol/L NaOH (panel E)

We also performed time-resolved small-angle X-ray scattering (TR-SAXS) experiments to monitor the de-oligomerization of (Uch37)n (where n is the number of Uch37 molecules in the oligomer) during its transition from an oligomer to a monomer in the presence of Rpn13C. The change in the values of I_0_ and R_g_ as a function of time (Fig. 3A and 3B) supports the following proposed mechanism for de-oligomerization of Uch37 by Rpn13C:

I. (Uch37)n + Rpn13C → (Uch37)n/Rpn13C, where n can be 2–4 and (Uch37)n/Rpn13C is an intermediate complex;
II. (Uch37)n/Rpn13C → Uch37-Rpn13C + (Uch37)n-1, where Uch37-Rpn13C is the complex of Uch37 and Rpn13C;
III. Uch37 + Rpn13C → Uch37-Rpn13C.

In this mechanism, steps I and II appear to occur concurrently until all Uch37 oligomers are consumed, whereas step III is a relatively slower step. An explanation for this mechanism can be formed based on the following observation. After the mixing of Uch37 with Rpn13C, the I_0_ of the system increased initially (<20 ms post mixing) compared to the I_0_ of a simple mixing case in which the samples were assumed to be physically mixed without any chemical reaction (Fig. 3A). A moderate increase in the I_0_ implied that Rpn13C interacted with the oligomerized Uch37 [(Uch37)n], forming a larger intermediate (Uch37)n/Rpn13C complex. During the same time interval (<20 ms), the R_g_ value of the system did not change significantly because the R_g_ values of (Uch37)n and (Uch37)n/Rpn13C are similar (Fig. 3B). The decrease in the apparent R_g_ and I_0_ in the range from 20 ms to 1 s mainly resulted from the repetitive processes of step II. That decrease followed by a slight increase in R_g_ and I_0_ during the 1–100 s range could have arisen from a situation in which (Uch37)n and (Uch37)n/Rpn13C were most likely all consumed, and step III became the major reaction process (Fig. 3A and 3B). Thus, the changes in R_g_ and I_0_ support the hypothesis that Rpn13C could disrupt the Uch37 oligomerization and form a heterodimer.

To further confirm the oligomerization of Uch37 in solution, assays based on fluorescence resonance energy transfer (FRET) were used to monitor both the oligomers of Uch37 in solution and their de-oligomerization by Rpn13C. Hetero-oligomers of Uch37 that were randomly labeled with Alexa Fluor 647 and Alexa Fluor 555 were formed as described in the Methods. FRET will occur when Alexa Fluor 647 and Alexa Fluor 555 are sufficiently close (<80 Å); the model, which is most likely to be tetrameric (Fig. S3), indicates that this distance requirement is met for Uch37 oligomers. As expected, we detected significant FRET for Uch37 (labeled with Alexa Fluor 647 or Alexa Fluor 555) alone at a concentration of 250 nmol/L (approximately 9.25 μg/mL) (Fig. 3C). The addition of Rpn13C, which monomerizes the Uch37, resulted in a decrease in FRET, as indicated by the decrease in the acceptor fluorescence at 664 nm and the corresponding increase in the donor fluorescence at 553 nm (Fig. 3C). Thus, the FRET assay further confirmed that the oligomerization of Uch37 and its de-oligomerization by Rpn13C even at extremely low concentrations.

To confirm that the interaction of Rpn13C with Uch37 was specific, we performed surface plasmon resonance (SPR) experiments and measured the affinity between Rpn13C and Uch37. Uch37 bound to Rpn13C tightly with a low dissociation constant (*K*_d_ = 5.35 nmol/L, Fig. 3D). Interestingly, 4 mol/L NaCl could not reverse the interaction between Uch37 and Rpn13C, indicating a role for hydrophobic interactions in the formation of the complex (Fig. 3E). Rpn13C could be dissociated from Uch37 in the presence of 15 mmol/L NaOH (Fig. 3E).

### Deletion of the C-terminal region de-oligomerized Uch37

**Figure 4:**
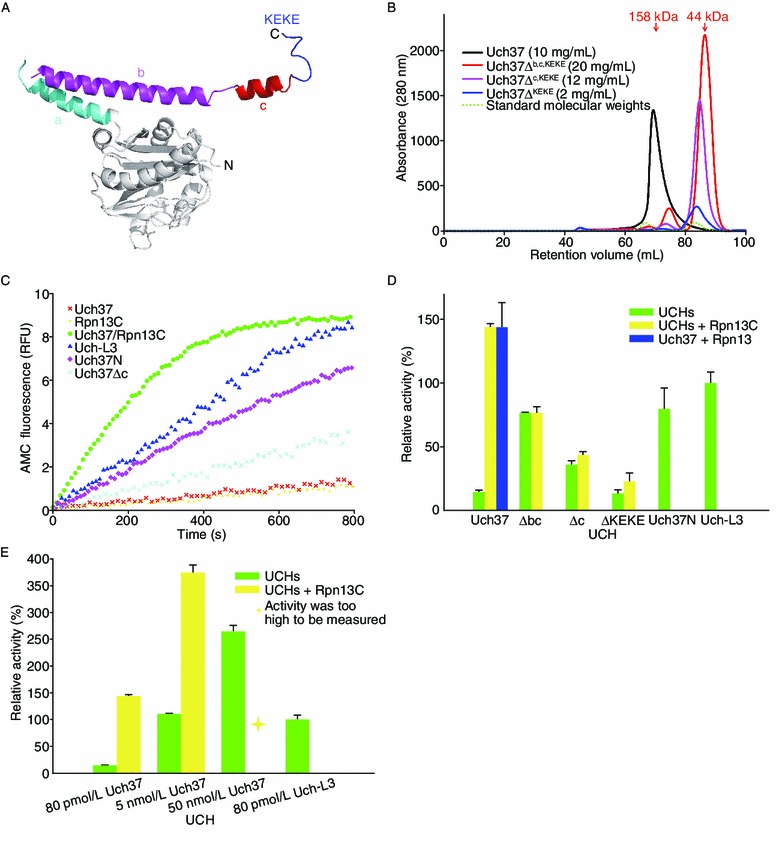
**Deletion of the C-terminal region de-oligomerized and activated Uch37**. (A) Cartoon of Uch37 depicting regions of the C-terminal extension region (helices a, b, and c and the KEKE motif). (B) Size exclusion profiles of the Uch37 truncations. The truncations of the C-terminal extension region had longer retention times than the full-length Uch37. Ovalbumin (44 kDa) and γ-globulin (158 kDa) were run as standards under identical conditions. The positions of the standards are marked with green arrows. (C–E) The activity of Uch37 in the presence or absence of the full-length Rpn13 or N-terminal truncated (Δ^1−269^) Rpn13C was measured using Ub-AMC as the substrate. The relative fluorescence units emitted by different constructs are shown as a function of time (C). Uch37 exhibited the highest activity in the presence of Rpn13. UCH-L3 was used as a control, with its activity considered to be 100 %. Uch37N, Δbc, Δc, and ΔKEKE represent the Uch37 catalytic domain, Uch37Δ^b,c,KEKE^, Uch37Δ^c,KEKE^, and Uch37Δ^KEKE^, respectively (D). (E) Uch37 at different concentrations could be activated by Rpn13C. The values are the average of three independent experiments; the error bars represent the standard error of the mean

The C-terminal extension region of Uch37 folds into three α-helices—helix a (Ha), residues 226–245; helix b (Hb), residues 256–288; and helix c (Hc), residues 291–303—followed by a long flexible region spanning residues 304–329 and containing the KEKE motif (Fig. 4A). All three helices have the potential to form a coiled-coil motif, which has been implicated in protein-protein interactions, particularly in the formation of multimeric complexes and molecular recognition (Zhang et al., 2009; Larzabal et al., 2010). SCORER 2.0 software identified that Hc formed perfect coiled-coil motifs (Armstrong et al., 2011). As predicted, four Hc helices interacted with each other mainly through hydrophobic residues, such as F294, I295, L298, and T301, as shown in Supplementary Fig. S2C.

To investigate the biological role of Hb, Hc, and the KEKE motif, three C-terminal truncations of Uch37, Uch37Δ^Hb,Hc,KEKE^, Uch37Δ^Hc,KEKE^, and Uch37Δ^KEKE^, were constructed (Fig. 1A). The oligomerization states of all three truncations were analyzed by SEC and AUC. The theoretical MWs calculated from the sequences were 29.1, 33.3, and 34.9 kDa for Uch37Δ^Hb,Hc,KEKE^, Uch37Δ^Hc, KEKE^, and Uch37Δ^KEKE^, respectively. Interestingly, at approximately 10 mg/mL, all three truncations eluted as single peaks in SEC using a 120 mL Superdex G200 column. The retention volumes were 86.46, 84.76, and 83.89 mL for Uch37Δ^Hb,Hc,KEKE^, Uch37Δ^Hc,KEKE^, and Uch37Δ^KEKE^, respectively (Fig. 4B). From the standard curve, the estimated MWs for Uch37Δ^Hb,Hc,KEKE^, Uch37Δ^Hc,KEKE^ and Uch37Δ^KEKE^ were 34.6, 39.4, and 43.7 kDa, respectively, suggesting that all three truncations existed as monomers. However, the full-length Uch37 had a shorter retention time (69.21 mL) and eluted as an oligomer (estimated MW of 128.9 kDa) when subjected to SEC under identical conditions (Fig. 4B). Next, we performed AUC analysis on Uch37Δ^Hc,KEKE^ and Uch37Δ^KEKE^ to further confirm the oligomerization states of the proteins. The root-mean-square deviation (r.m.s.d.) of the best fitting to the continuous size distribution model was 0.0072 for Uch37Δ^c,KEKE^ and 0.0070 for Uch37Δ^KEKE^ (Table S2). Sedimentation velocity experiments revealed that the Uch37Δ^Hc, KEKE^ truncation had an f/f0 ratio of 1.24 (values near 1.2 correspond to globular shapes) and exists as a monomer (estimated 30.1 kDa MW from AUC), whereas Uch37Δ^KEKE^ and the WT Uch37 exist as a mixture of different oligomers in solution (Figs. 1E and S4). The observation that Uch37Δ^KEKE^ eluted as a single peak at a position close to that of the monomer of Uch37 in SEC but behaved as a mixture of oligomers in AUC was intriguing. The deletion of the KEKE motif may have altered the surface properties of the protein, and therefore, Uch37Δ^KEKE^ could have oligomerized. Currently, we do not have a good explanation for this perplexing behavior of Uch37Δ^KEKE^. In the crystal structure of Uch37 (PDB ID 3IHR), the Uch37 molecules are packed as a tetramer even though the KEKE motif is not visible. Therefore, it is likely that the KEKE motif may not play an essential role in the oligomerization of Uch37.

Taken together, the crystal packing analysis of Uch37 (PDB ID 3IHR and 3A7S) and the SEC and AUC analyses of different C-terminal truncations of Uch37 suggest that the C-terminal extension region, particularly residues 291–303, play an important role in the oligomerization of the protein in solution. The coiled-coil interactions of Hc form the basis for the packing of Uch37 as a tetramer (Fig. S2C). The binding of Rpn13 to the KEKE motif of Uch37 likely perturbs the hydrophobic interactions of tetrameric Hc, resulting in disassembly of the oligomer into a monomer via the formation of a stable Rpn13-Uch37 complex that provides a means to keep Uch37 in the monomeric state.

### Reconstruction of the Uch37-Rpn13C complex structure by SAXS

**Figure 5:**
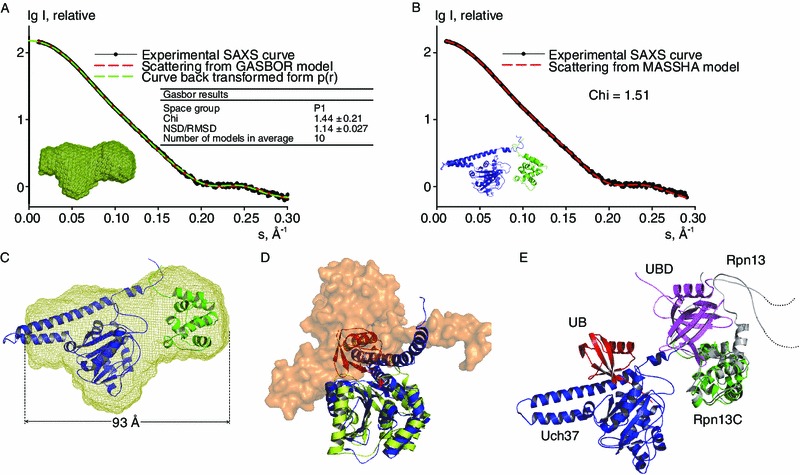
**Rpn13-activated Uch37 by reversing the oligomerization, as revealed by an SAXS model**. (A) Model generated by GASBOR for the Uch37-Rpn13C complex in solution. The graph illustrates that the model fit the experimental data well. (B) Model of the Uch37-Rpn13C complex generated by MASSHA. The graph illustrates that the model fit the experimental data well. (C) A low-resolution envelope of the Uch37-Rpn13C complex with the model fit to the envelope. (D) Dimer of Uch37 displaying one chain in a surface representation, with the second chain is shown as a cartoon (blue). Complex of UCH-L3 (light green) with Ub (red) superimposed over the dimer of Uch37 to depict the steric hindrance preventing access to the Uch37 active site. (E) The Rpn13-Uch37-Ub hetero-trimeric model generated by aligning UCH-L3-Ub (PDB ID 1XD3) and Rpn13 (PDB ID 2KR0) to the Uch37-Rpn13C complex structure obtained by MASSHA. The steric block is removed when Uch37 associates with Rpn13C (shown as a green cartoon). The ubiquitin-binding domain (UBD) of Rpn13 (violet magenta) sits next to its Uch37-binding domain (grey) at a position approximately adjacent to that of Ub such that the UBD could anchor the ubiquitins that precede the terminal ubiquitin during the catalysis. The Uch37-binding surface of Rpn13C (yellow) is facing the Uch37 molecule

To visualize the interaction between Uch37 and Rpn13C, we employed *ab initio* and rigid body modeling methods to construct the Uch37-Rpn13C complex from the SAXS data (Fig. 5). Ten individual GASBOR calculations without symmetry restraint (P1) (Svergun et al., 2001) were performed to construct a model for the Uch37-Rpn13C complex. The resultant model of the Uch37-Rpn13C complex had an acceptable chi value of 1.44 ± 0.21 (Fig. 5A). The consensus shape of the Uch37-Rpn13C complex mainly contains two parts—Uch37 (left part) and Rpn13C (right part) (Fig. 5A). In rigid body modeling, a Uch37 monomer model (PDB ID 3IHR) and the NMR structure of Rpn13C were modeled together using MASSHA (Konarev et al., 2001) (Fig. 5B). Considering the flexibility of the KEKE motifs, the final chi value, 1.50, is considered reasonable. Thus, we obtained a model that is consistent with the biochemical knowledge that the KEKE motifs of Uch37 and of Rpn13C associate to form a heterodimer (Fig. 5B and 5C). Furthermore, the atomic model of the Uch37-Rpn13C complex generated by MASSHA could fit the *ab initio* low-resolution shape well (Fig. 5C). In this SAXS-based Uch37-Rpn13C complex model, the Uch37-binding surface of Rpn13C reported by Chen et al. was facing toward the Uch37 molecule (Fig. 5D). Although the overall SAXS-based Uch37-Rpn13C complex model is in agreement with the model reported by Chen et al., the distance between the Uch37-binding surface of Rpn13 and the Uch37 molecule is slightly larger. One possible reason for this difference might lie in the flexibility of the SAXS model.

### Auto-inhibition of Uch37 and its activation by Rpn13C

To determine why Uch37 exhibited low activity against the Ub-AMC substrate, which could be hydrolyzed by Uch37 homologs (Fig. 4C), we aligned the structure of the catalytic domain of Uch37 with those of UCH-L1, UCH-L3, and YUH1. This analysis revealed that the Uch37 catalytic domain was highly conserved and contained all of the components necessary for the catalysis. Because the catalytic domain did not provide sufficient clues about its mechanism of auto-inhibition, we examined the C-terminal extension region of Uch37 for its potential role in auto-inhibition. In solution, Uch37 existed mainly as oligomers that were primarily mediated by the C-terminal extension region. In the presence of Rpn13C, Uch37 formed a 1:1 complex with Rpn13C. Such a configuration of Uch37 in the presence of Rpn13C readily cleaved the substrate Ub-AMC (Fig. 4C). Thus, the Uch37 oligomerization mediated by the C-terminal extension region maintained the auto-inhibition of the enzyme.

To delineate the structural basis of the auto-inhibition of Uch37 and its activation by Rpn13C, we used a Uch37 dimer model derived from its crystal structure and compared it with the structure of the binary complex of ubiquitin-bound Uch37 (PDB ID 4IG7) and its homolog UCH-L3 (PDB ID 1XD3). These comparisons revealed that the position of Ub is occupied by another molecule in the dimeric Uch37 models, preventing access to the active site (Fig. 5E). Thus, in its auto-inhibited form, a second molecule of Uch37 sterically obstructs the entry of a substrate, such as Ub-AMC, into the active site. Rpn13C released Uch37 from the inactive oligomeric state by forming a 1:1 complex with Uch37. Rpn13 eliminated the steric hindrance and provided access to the active site of Uch37, resulting in the activation of Uch37 (Fig. 5D).

The structure of the full-length Rpn13 (PDB ID 2KR0) contains a Pru domain sitting next to Rpn13C (Chen et al., 2010). Structural superimposition of the full-length Rpn13 over the heterotrimeric Uch37-Rpn13C-UB model revealed that the Pru domain of Rpn13 might sit at a position approximately adjacent to that of Ub. Such an arrangement of the proteins implied a potential role of the Pru domain in anchoring the ubiquitins that precede the distal Ub during the catalysis by Uch37.

### Deletion of the C-terminal region-activated Uch37

To further demonstrate that Rpn13C activated Uch37 through de-oligomerization, we performed Ub-AMC activity assays for Uch37 and its C-terminal region deletion truncations in the presence or absence of Rpn13. Initial optimization studies on the assay conditions revealed that Uch37 alone had some limited activity (Fig. 4D). However, this activity was very low when compared with the activity of the Uch37 catalytic domain alone (14.31 ± 1.65 versus 79.55 ± 16.59, considering the activity of UCH-L3 to be 100%) (Fig. 4D). Concentration-dependent activity assays revealed that as the concentration of Uch37 alone increased, the residual activity of Uch37 also increased, with relative activities of 14.31 ± 1.65, 110.31 ± 1.37, and 264.09 ± 11.62 in 80 pmol/L, 5 nmol/L, and 50 nmol/L, respectively. However, the addition of Rpn13 to the assays resulted in a dramatic increase in the enzyme activity of Uch37 (143.82 ± 2.63 in 80 pmol/L; 374.06 ± 13.85 in 5 nmol/L; the relative activity in 50 nmol/L was too high to be measured) (Fig. 4E). This increase in activity was a function of the enzyme concentration. As expected, all three C-terminal truncations of Uch37 (all without the KEKE motif) could not be activated any further by Rpn13C (Fig. 4D). This result is consistent with the inference derived from the biophysical characterization of the Uch37-Rpn13C complex that Rpn13 activates Uch37 through its interaction with the C-terminal domain of Uch37, particularly the KEKE motif. Furthermore, Uch37Δ^Hb,Hc,KEKE^ and Uch37Δ^Hc,KEKE^ displayed greater de-ubiquitinating activities than did Uch37Δ^KEKE^ (76.41 ± 0.56 and 35.91 ± 3.09 versus 13.32 ± 2.69), which displayed an activity that was similar to that of the auto-inhibited WT Uch37 (14.31 ± 1.65). Uch37Δ^Hb,Hc,KEKE^ exhibited comparable activity to that of the UCH domains, such as UCH-L3 and was roughly twice as high as that of Uch37Δ^Hc,KEKE^. This finding indicates that both Hb and Hc play critical roles in the auto-inhibition of Uch37. However, it can’t be excluded that Hb helix itself probably inhibit the activity in other unexpected way. Taken together, these activity assay results lend direct support to the conclusion that the auto-inhibition of Uch37 is a result of its oligomerization, which is shed on the presence of Rpn13.

Despite the multiple lines of evidence presented here, the fact that the activity of Uch37 in the presence of Rpn13C is considerably higher than that of the Uch37 catalytic domain alone (143.82 ± 2.63 versus 79.55 ± 16.59) suggests that the hypothesis—Rpn13 releases the oligomerization of Uch37, resulting in its activation—can partly explain the mechanism of Uch37 activation. There are likely other factors that contribute to the activation of Uch37 by Rpn13. For example, Rpn13C could possibly stabilize the Uch37 active-site cross-over loop, enhancing its activation. In addition, the full-length Rpn13 or Rpn13 anchored on 19S could bind, stabilize, and optimally orient poly-ubiquitin chains for Uch37. However, the basis for any enhancement in the activity of Uch37 lies in the reversal of its oligomerization, a process that is mediated by Rpn13.

## Conclusions

Rpn13, which functions as a ubiquitin receptor, and the de-ubiquitinating enzyme Uch37 are an integral part of the 26S proteasome. Uch37 by itself has very low de-ubiquitinating activity. Our analyses of the solution status of Uch37 explained the possible reasons behind this auto-inhibition of Uch37. Uch37 could form oligomers in solution. Such an oligomerization of Uch37 was mainly mediated by the coiled-coil motif of Hc. When in an oligomeric state, the C-terminal extension region from the other monomer covered and sterically obstructed the placement of ubiquitin into the active site of another molecule of Uch37. Using a combination of mutagenesis, biochemical, and small-angle X-ray scattering (SAXS) techniques, we demonstrated that Rpn13 activated the auto-inhibited Uch37 by de-oligomerizing it and sequestering it to form a 1:1 stoichiometric complex. The findings help to understand the mechanism of Uch37 auto-inhibition clearly and shed light on the activation of Uch37 by Rpn13.

## Materials and Methods

### Plasmid construction

The full-length Uch37 gene was amplified from a plasmid containing human Uch37 (a gift from Professor Robert E. Cohen) (Yao et al., 2006). The resultant gene fragment was ligated into pMCSG7 encoding His-TEV-Uch37 as previously reported (Niu et al., 2013). Truncations and mutations were designed based on this plasmid pMCSG7-Uch37 using a Quik Change^TM^ site-directed mutagenesis kit following the manufacturer’s instructions (Stratagene, La Jolla, CA, USA). The gene encoding full-length Rpn13 was synthesized by Sangon Biotech (Shanghai) Co., Ltd and was cloned into pET-28b using *Bam*H I and *Xho*I. Truncations of Rpn13 were cloned into pMCSG7 using the method described above. All the plasmids were sequenced to verify the sequence.

### Protein expression and purification

The plasmids harboring the target genes were transformed into *E. coli* BL21(DE3). The cells were grown in Luria-Bertani (LB) medium containing ampicillin (100 μg/mL) at 37°C until the OD_600_ reached 0.8. The culture was then induced with 0.2 mmol/L isopropyl-β-D-thiogalactoside (IPTG) for 20 h at 16°C. Cells were harvested by centrifugation, lysed by sonication, and clarified by centrifugation; the supernatant was then applied to a nickel-nitrilotriacetic acid (Ni-NTA) resin gravity column (Qiagen, Valencia, CA, USA) that had been previously equilibrated with PBS (137 mmol/L NaCl, 2.7 mmol/L KCl, 50 mmol/L Na_2_HPO4, and 10 mmol/L KH_2_PO4 at pH 7.4). The column was first washed with 100 mL of PBS, then washed with 100 mL of PBS containing 20 mmol/L imidazole, and finally eluted with PBS containing 300 mmol/L imidazole. After buffer exchange, the His-tag was cleaved by a tobacco etch virus (TEV) treatment. Uncut protein was separated by a second Ni-affinity chromatography. Fractions containing the protein were pooled, concentrated, and loaded on a Superdex G200 size exclusion chromatography column (Amersham) equilibrated with 20 mmol/L Tris-HCl at pH 8.0, 200 mmol/L NaCl, and 2 mmol/L DTT. Pure proteins, including Uch37 and Rpn13C, were pooled and stored at −80°C. Uch37 and Rpn13C were mixed at a ratio of 1:2. The mixture was loaded onto a Superdex G200 column again to separate the Uch37-Rpn13C complex from excess Rpn13. The resulting Uch37-Rpn13C complex was stored at −80°C until further use.

### Size exclusion chromatography (SEC)

Protein samples (approximately 10 mg/mL) were loaded onto a Superdex G200 column (Amersham) equilibrated with 20 mmol/L Tris-HCl at pH 8.0, 200 mmol/L NaCl, and 2 mmol/L DTT. The elution volumes (V_e_) were recorded. In the Superdex G200 (120 mL) column (void volume V_0_ = 45.2 mL), ovalbumin (44 kDa) and γ-globulin (158 kDa) eluted at 82.87 and 66.69 mL, respectively. The standard curve was obtained by the linear correlation between log_10_MW and V_e_/V_0_. The MWs for the proteins and their complexes were calculated from Ve based on the standard curve.

### Analytical ultracentrifugation (AUC) analysis

Analytical sedimentation velocity experiments were conducting using a ProteomeLab™ XL-I system (Beckman Coulter) according to our previous report (Ouyang et al., 2012). Briefly, an An-60Ti rotor was used to centrifuge protein samples suspended in 20 mmol/L Tris-HCl at pH 8.0, 200 mmol/L NaCl, and 2 mmol/L DTT at 60,000 rpm with an A_280nm_ of approximately 0.8. The absorbance was read at 280 nm. A set of 93 scans were collected at 1 min intervals. Sedfit software was used for size distribution analysis with a continuous c(s) distribution model with the default parameters (Schuck, 2000). After interpretation and refinement of the results, the distribution was displayed and exported with a confidence level (F-ratio) of 0.9. The statistics for the AUC runs are listed in Table S2. The MWs of the globular proteins were obtained by converting the S values with the f/f0 ratio in Sedfit. The percentage distribution of each peak was obtained by S peak integration in Sedfit.

### NMR studies on Rpn13C

The plasmid pMCSG7-Rpn13C was transformed into *E. coli* BL21(DE3). Cells were grown in M9 medium containing glucose (0.2% *M*/*V*), MgSO_4_ (1 mmol/L) and ampicillin (100 μg/mL). ^15^N ammonium chloride and/or ^13^C glucose was used for isotope labeling. Labeled Rpn13C was purified using the same procedure as for the native one. The NMR samples of Rpn13C contained approximately 0.5–1.0 mmol/L ^15^N- and ^13^C- uniformly labeled protein in 50 mmol/L phosphate sodium buffer at pH 6.0, 50 mmol/L NaCl, 0.02% (*w/v*) sodium 2,2-dimethylsilapentane-5-sulfonate (DSS), and 10% (*v/v*) ^2^H_2_O. All NMR experiments were performed at 298 K on a Bruker 600 MHz NMR spectrometer equipped with a z-gradient triple-resonance cryoprobe. Experiments using 2D ^1^H-^15^N and ^1^H-^13^C HSQC, 3D HNCA, CBCA(CO)NH, HNCACB, HNCO, HN(CA)CO, HBHA(CO)NH, HBHANH, HCCH-TOCSY, and CCH-TOCSY were performed for the backbone and side chain assignments of Rpn13C. Additionally, 3D ^1^H-^15^N and ^1^H-^13^C NOESY-HSQC spectra with mixing times of 120 ms were collected to generate distance restraints. All data were processed with Felix (Accelyris Inc.) and analyzed with NMRViewJ (Johnson and Blevins, 1994). The proton chemical shifts were referenced to the internal DSS, and the ^15^N and ^13^C chemical shifts were referenced indirectly (Markley et al., 1998). The structures of Rpn13C were initially calculated with the program CYANA (Güntert et al., 1997) and then refined using CNS (Brunger et al., 1998), whereas the manual assignments and semi-automated NOE assignments were performed using SANE (Duggan et al., 2001). Backbone dihedral angle restraints obtained using TALOS+ (Shen et al., 2009), as well as hydrogen-bond restraints based on the regular secondary structure patterns, were also incorporated into the structural refinement. From 100 initial structures, the 50 lowest-energy conformers of Rpn13C were selected for water-refinement using CNS and RECOORD Script (Nederveen et al., 2005), and the 20 lowest-energy conformers were selected to represent the final ensemble of structures for Rpn13C. The quality of the structures were analyzed using MOLMOL (Koradi et al., 1996) and PROCHECK-NMR (Laskowski et al., 1996).

### SAXS data collection and analysis

SAXS images were collected at the SIBYLS beamline (Advanced Light Source, Lorenz Berkeley National Lab, USA). Each sample was measured with three exposures, 0.5, 1, and 6 s, at 10°C at three concentrations. Data for Uch37 were collected at 1.25, 2.5, and 5 mg/mL in a buffer composed of 20 mmol/L Tris-HCl at pH 8.0, 200 mmol/L NaCl, and 2 mmol/L DTT, whereas data for Uch37-Rpn13C were collected at 1.5, 3, and 6 mg/mL. The scattering intensity I(Q) was measured for Q values (Q = 4πsinθ/λ, where 2θ is the scattering angle) ranging from 0.01 to 0.3 Å^−1^. The resulting scattering curves for each sample were radially averaged, and the buffer was subtracted. The data were integrated, scaled, and buffer subtracted to obtain the standard scattering curves. Multiple curves with different concentrations and different exposure times were scaled and averaged to generate the average scattering curve.

The initial R_g_ values from the Guinier plot analysis were analyzed by PRIMUS (Konarev et al., 2003). The P(r) distribution function was calculated with the program GNOM (Svergun, 1992). The MW was estimated directly using the P(r) distribution function from the web server (http://www.if.sc.usp.br/~saxs/saxsmow.html) (Fischer et al., 2010).

Low-resolution shape reconstructions were modeled by GASBOR (Svergun et al., 2001) from the calculated P(r) distribution curve. Uch37 and Rpn13C were modeled together based on the scattering curve using MASSHA (Konarev et al., 2001). Based on the KEKE interaction between Uch37 and Rpn13C, the position of Uch37 monomer was fixed, whereas Rpn13C was able to move freely around Uch37 with the constraint that the distance of the KEKE motifs was no larger than 7 Å. The high-resolution models were superimposed onto the low-resolution shape model by SUPCOMB (Kozin and Svergun, 2001).

### Time-resolved small angle X-ray scattering (TR-SAXS)

The steady-state small-angle X-ray scattering (SAXS) experiments were performed at beamline 12ID-B of the Advanced Photon Source (APS) at Argonne National Laboratory (Chicago site). The wavelength, λ, of the X-ray radiation was set to 1.033 Å. The scattered X-ray intensities were measured using a Pilatus 2M detector (DECTRIS Ltd). The sample-to-detector distance was set such that the detecting range of the momentum transfer, q [= 4π sinθ/λ, where 2θ is the scattering angle], of the SAXS experiments was 0.008–0.92 Å^−1^. To reduce the radiation damage, a flow cell made of a cylindrical quartz capillary with a diameter of 1.5 mm and a wall of 10 μm was used, and the exposure time was set to 1–2 s. To obtain good signal-to-noise ratios, 20 images were taken for each sample and buffer. The 2-D scattering images were converted to 1-D SAXS curves through azimuthal averaging after solid-angle correction and then normalization with the intensity of the transmitted X-ray beam using the software package developed at beamline 12ID-B. The time-resolved solution SAXS experiments were performed using a Bio-Logic SFM-400 stopped-flow mixer coupled to a Pilatus 100K detector (DECTRIS Ltd) at BioCAT beamline 18ID at APS. The Uch37 and Rpn13C were mixed at a 1:1 monomer molar ratio with an overall protein concentration of 3.7 mg/mL. The reaction was measured in the time range from 8 ms to 600 s. Two parameters, I_0_ and R_g_, were calculated for the system from the Guinier equation [I(q) = I_0_*exp(−R_g_^2^*q^2^/3)] and plotted against the time elapsed after mixing the two proteins. For a single-component system, I_0_ is the forward scattering, a direct indicator of the molecular weight, whereas R_g_ can provide information about the overall size of a molecule. For a system with multiple (N) molecular components, the apparent I_0_ and R_g_ can be written as follows: ${\text{I}}_{0} = \mathop \sum \nolimits_{{{\text{j}} = 1}}^{\text{N}} {\text{I}}_{{0{\text{j}}}}$*I*_0_ = ∑ _*j*=1_^*N*^*I*_0*j*_ and $R_{g}^{2} = \mathop \sum \nolimits_{j = 1}^{N} \frac{{I_{0j} R_{gj}^{2} }}{{I_{0} }}$, where *I*_0*j*_ [=*kC*_*j*_*MW*_*j*_], and *R*_*gj*_ are the parameters for the *j*th component, *C*_*j*_ is its concentration in terms of the mass, and *k* is a constant.

### Uch37 activity assay

Activity measurements were conducted with 80 pmol/L UCH enzyme and 0.5 μmol/L Ub-AMC (Sigma) as the substrate in the assay buffer (50 mmol/L Tris-HCl at pH 8.0, 0.5 mmol/L EDTA, 1 mmol/L DTT, and 0.1% BSA) at 25°C. For comparison, the activities for UCH-L3 and Uch37 with or without Rpn13 were measured simultaneously. To fully release the activity, Rpn13 was added to Uch37 in an excess molar amount. The fluorescence signals of AMC (excitation, 380 nm; emission, 460 nm) were monitored using a VarioskanFlash *(*Thermo Scientific*)* within 30 min. The velocities were determined by fitting the initial linear data points to a least-squares regression line with *R*^2^ scores greater than 0.95. For each sample, the activity assay was repeated at least three times for statistical analysis.

### Fluorescence resonance energy transfer (FRET)

Full-length Uch37 containing only one cysteine (C27) was obtained by four rounds of QuikChange site-directed mutagenesis. C9, C88, C100, and C191 were progressively mutated into alanine. The Uch37 containing only one cysteine (C27) was expressed and purified using the procedure mentioned previously. Purified Uch37 (C27 only) was incubated with a five-fold molar excess of dye (Alexa Fluor 555 and Alexa Fluor 647, Invitrogen) for 5 h at 16°C. The reaction was quenched with a 10-fold excess of DTT over the dye for 1 h at 16°C, and the free dye was separated by gel filtration. FRET was measured on a VarioskanFlash (Thermo Fisher Scientific) using 100 μL of labeled Uch37 (250 nmol/L). An excess molar amount of Rpn13C was added, the mixture was incubated for 30 min, and the FRET signals were measured.

### Surface plasmon resonance (SPR)

SPR experiments were performed in duplicate using a Biacore3000 instrument (Biacore, Inc.) at 25°C in PBS running buffer. Rpn13C was immobilized on CM5 chips following the standard amine coupling procedure recommended by the manufacturer. For kinetic measurements, sensorgrams were recorded by passing 60 μL of various concentrations of Uch37 over the immobilized Rpn13C surface at a flow rate of 30 μL/min with a 2 min association phase followed by a 10-min dissociation phase. The surface was regenerated between each experiment with two consecutive injections (0.5 min) of 15 mmol/L NaOH at 30 μL/min followed by a 1 min equilibration phase in the PBS buffer before the subsequent experiment. Identical injections over blank surfaces were subtracted from the data for kinetic analysis. The binding kinetics were analyzed by BIAevaluation software (Biacore) using a 1:1 Langmuir binding model. All injections were performed in duplicate and yielded highly similar results.

## Accession Code

The atomic coordinate and structure factor of Rpn13C were deposited in the Protein Data Bank (www.pdb.org) and the PDB code is 2MKZ and the BMRB accession ID is 19801.

## Electronic supplementary material

Below is the link to the electronic supplementary material.

Supplementary material 1 (PDF 1137 kb)

## Abbreviations

AUC: analytical ultracentrifugation
CP: core particle
DUB: de-ubiquitinating enzyme
FRET: fluorescence resonance energy transfer
Pru: pleckstrin-like receptor for the Ub
RP: regulatory particle
SAXS: small-angle X-ray scattering
SEC: size exclusion chromatography
SPR: surface plasmon resonance
TR-SAXS: time-resolved SAXS
Ub: ubiquitin
Ub-AMC: ubiquitin-7-amino-4-methylcoumarin
UCH: ubiquitin C-terminal hydrolase
