# Supplementary material for: Mechanism of the Rpn13-induced activation of Uch37
Source: Protein Cell. 2014 Apr 22;5(8):616–30. doi: 10.1007/s13238-014-0046-z (PMC4130924; doi:10.1007/s13238-014-0046-z)
Supplement: Supplementary file 1 — Supplementary material 1 (PDF 1137 kb) [file 13238_2014_46_MOESM1_ESM.pdf]

Mechanism of the Rpn13-induced activation of Uch37

Lianying Jiao[1]‡, Songying Ouyang[1]‡, Neil Shaw[1], Gaojie Song[2], Yingang Feng[3], Fengfeng Niu[1], Weicheng Qiu[1], Hongtao Zhu[1], Li-Wei Hung[4], Xiaobing Zuo[5], Eleonora Shtykova V.[6], Ping Zhu[1], Yu-Hui Dong[7], Ruxiang Xu[8]*, Zhi-Jie Liu[1,3]*

[1]National Laboratory of Biomacromolecules, Institute of Biophysics, Chinese Academy of Sciences, Beijing 100101, China
[2]iHuman Institute, ShanghaiTech University, Shanghai 201210, China
[3]Qingdao Institute of Bioenergy and Bioprocess Technology, Chinese Academy of Sciences, Qingdao 266101, China
[4]Physics Division, Los Alamos National Laboratory, Los Alamos, NM 87545, USA
[5]X-ray Science Division, Argonne National Laboratory, 9700 South Cass Avenue, Argonne, IL 60439, USA
[6]Institute of Crystallography, Russian Academy of Sciences, 59 Leninsky Pr., 117333 Moscow, Russian Federation
[7]Center for Multi-disciplinary Research, Institute of High Energy Physics, Chinese Academy of Sciences, Beijing 100049, China
[8]Department of Nerosurgery, The Military General Hospital of Beijing PLA, Beijing 100700, China
*E-mails to be used for correspondence: zjxuruxiang@163.com; zjliu@ibp.ac.cn

‡These authors contributed equally to this work.

Supplementary Figures S1-S5 and Tables S1-S2

Supplementary Figure S1. Size exclusion chromatography of Uch37 at different concentrations.

Supplementary Figure S2. Structure of Uch37.
(A) Monomer of Uch37. (B) Inspection of the symmetry mates revealed that Uch37 crystallized as a tetramer. (C) Coiled-coil interaction of helix c (Hc) holding the dimers of Uch37 together, resulting in the formation of a tetramer.

Supplementary Figure S3. Mutagenesis of Uch37 for FRET analysis.
Except for Cys27, all other cysteine residues (Cys9, Cys88, Cys100, and Cys191) were mutated to alanine. The distance between any two Cys27 of monomers was < 80 Å and was therefore suitable for FRET analysis.

Supplementary Figure S4. Analytical ultracentrifugation analysis of Uch37, truncations, and the Uch37-Rpn13C complex. (A) Analytical ultracentrifugation analysis of Uch37Δ^{KEKE}. Because oligomerization of Uch37 occurs via the C-terminal extension region, deletions of the C-terminal region were examined for the role of those regions in oligomerization. The C-terminal extension region contains helices a, b, and c and the KEKE motif. Removal of the KEKE motif does not improve the homogeneity of the protein, as indicated in the ultracentrifugation analysis above. A majority of the Uch37Δ^{KEKE} is oligomeric in solution. (B) Analytical ultracentrifugation analysis of Uch37$\Delta^{Hc,\ KEKE}$. Deletion of the region after helix b (Uch37$\Delta^{Hc,\ KEKE}$) results in a homogenous monomeric protein. Thus, C-terminal helix c and the KEKE motifs are important for the oligomerization of Uch37 and auto-inhibition.

Supplementary Table S1. Experimental restraints and structural statistics for the 20 lowest-energy structures of Rpn13C.
Supplementary Table S2. Statistics for AUC experiments.

Supplementary Figure S1

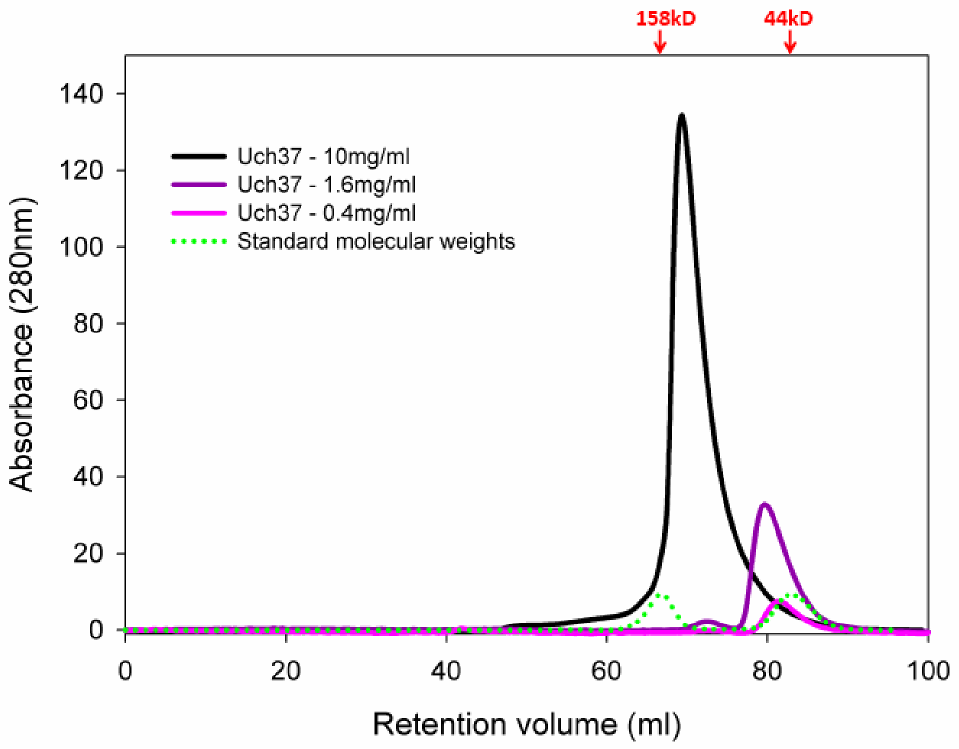

Size exclusion chromatography of Uch37 (200 μl) at different concentrations revealed concentration dependent oligomerization of the protein. The results are in agreement with those previously reported by Burgie *et al*. Position of molecular weight standards was indicated by arrows.

Burgie, S. E., Bingman, C. A., Soni, A. B. & Phillips, G. N., Jr. (2011). *Proteins* **80**, 649-654.

Supplementary Figure S2

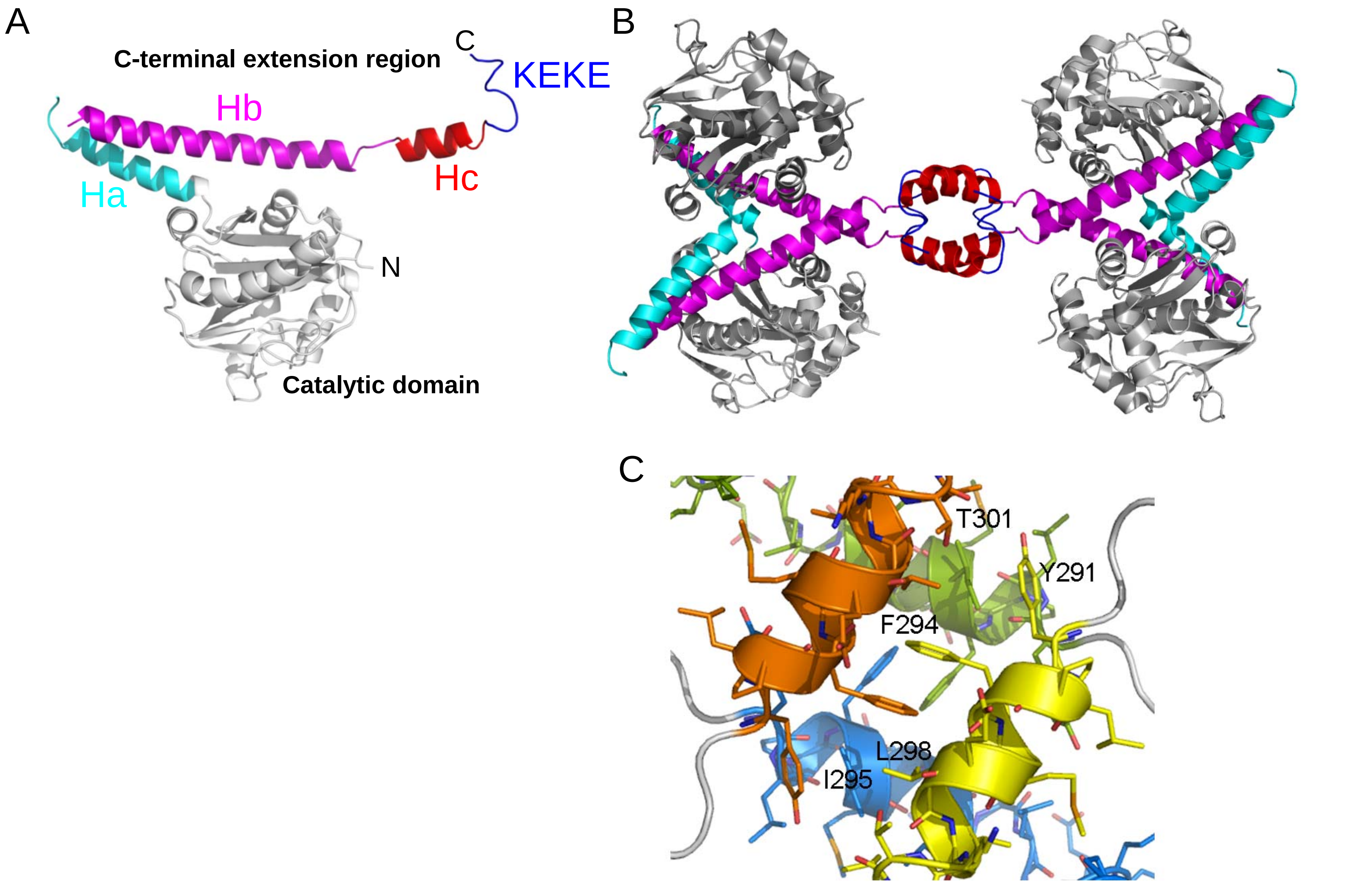

Structure of Uch37 (PDB_ID 3IHR).
(A) Uch37 monomer
(B) Inspection of symmetry mates reveals that Uch37 had crystallized as a tetramer
(C) Coiled coil interaction of Hc holding the dimers of Uch37 together, resulting in the formation of tetramer

Supplementary Figure S3

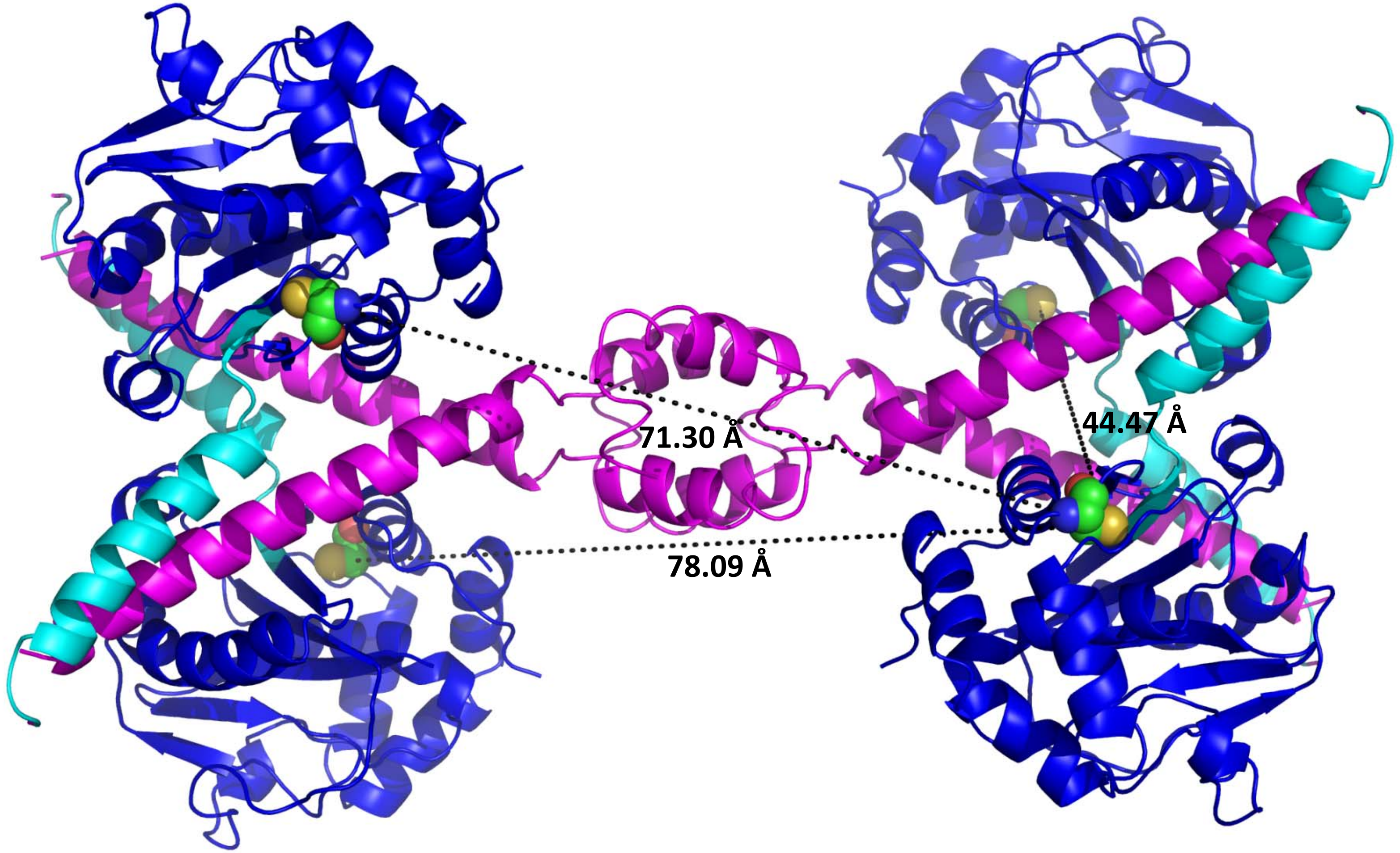

Mutagenesis for FRET analysis.
Cartoon of Uch37 in solution depicting distances between the Cys27 of the monomers within the tetramer. Cysteines are represented as spheres. Except for Cys27, all other cysteine residues (Cys9, Cys88, Cys100, and Cys191) were mutated to alanine. The distance between any two Cys27 of monomers is < 80 Å and therefore suitable for FRET analysis.

Supplementary Figure S4

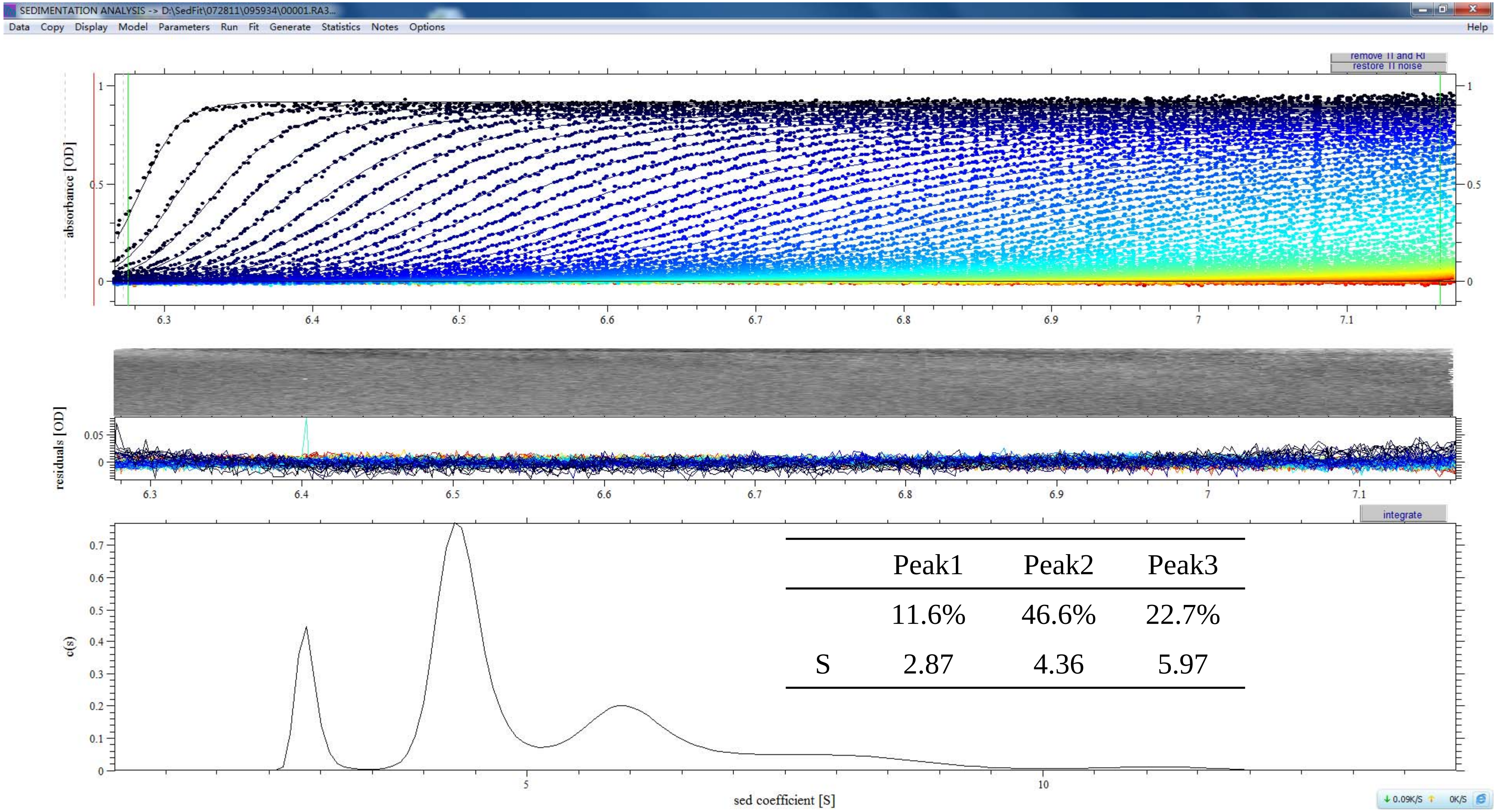

	Peak1	Peak2	Peak3
	11.6%	46.6%	22.7%
S	2.87	4.36	5.97

(A) Analytical ultra centrifugation of Uch37 Δ^{KEKE}

Since oligomerization of Uch37 occurs via the C-terminal extension region, deletions of the C-terminal region were examined for their role in oligomerization. The C-terminal extension region contains helices a, b, c, and the KEKE motif. Removal of the KEKE motif (B) does not improve the homogeneity of the protein as indicated in the ultracentrifugation analysis above. A majority of the Uch37Δ^{KEKE} is oligomeric in solution.

Supplementary Figure S4 (continued)

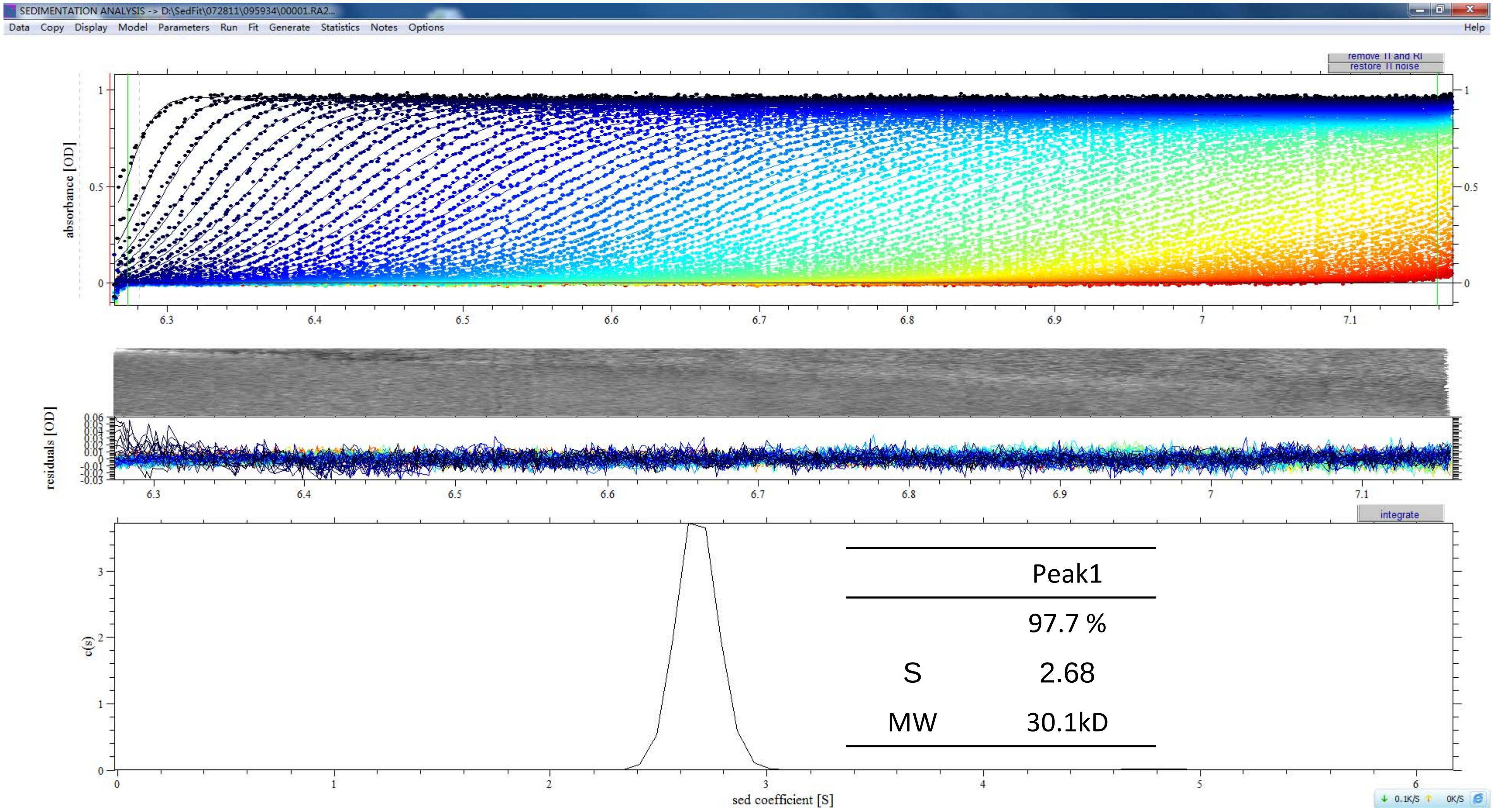

(B) Analytical ultra centrifugation of Uch37Δ $^{Hc, KEKE}$

Deletion of the region after helix b (Uch37Δ $^{Hc, KEKE}$) results in a homogenous monomeric protein. Thus C-terminal helix c and KEKE motifs are important for oligomerization of Uch37 and auto-inhibition.

Supplementary Table S1 Experimental restraints and structural statistics for the 20 lowest energy structures of Rpn13C.

Distance restraints	
Intra-residue	700
Sequential	536
Medium	355
Long range	310
Ambiguous	1901
Total	3802
Hydrogen bond restraints	86
Dihedral angle restraints	
ϕ	88
ψ	88
Total	176
Violations	
Max. NOE violations (Å)	0.183
Max. torsion angle violations (°)	1.92
Deviations from idealized geometry	
Bond lengths (Å)	0.0094 ± 0.0003
Bond angles (°)	1.087 ± 0.039
Improper (°)	1.397 ± 0.101
PROCHECK statistics (%)	
Residues in most favoured regions	81.1
Residues in additionally allowed regions	14.5
Residues in generously allowed regions	1.7
Residues in disallowed regions	2.7
RMSD for averaged model (Å)	
Backbone heavy atoms	
All residues [a]	0.36 ± 0.07
Regular secondary structures [b]	0.33 ± 0.06
All heavy atoms	
All residues [a]	0.68 ± 0.05
Regular secondary structures [b]	0.69 ± 0.05

[a] Residues used to calculate RMSD values of all residues include 288-384.
[b] Regular secondary structure regions are residues 288-291, 294-301, 304-313, 324-330, 334-349, 354-359, 363-371, and 374-384.

Supplementary Table S2 Statistics from AUC experiments

	Uch37	Uch37/Rpn13C	Uch37 $\Delta^{Hc,KEKE}$	Uch37 Δ^{KEKE}
ME regularization			P = 0.90	
Fric ratio	1.00599	1.57713	1.24042	1.06101
rmsd	0.00987	0.01133	0.00715	0.00700
Run test Z	30.83	27.54	14.53	25.55

Goodness-of-fit can be judged by rmsd and Run test Z score. Smaller the value, better the fit. The recommended upper limit of “Runs test Z” is 30. For non-globular sample, Run’s test Z may be larger. Rmsd could also be used for judging the quality of fittings.
